# Clinical value of a whole blood interferon-γ release assay for the diagnosis of *Mycobacterium tuberculosis* infection during antitubercular treatment

**DOI:** 10.3892/etm.2013.1127

**Published:** 2013-05-21

**Authors:** HAIFENG WANG, ZHENYUN LI, QUNMEI ZHANG, GUANGJIAN LU, QINGJIANG WANG, LIGONG ZHANG, CHENGUANG ZHANG

**Affiliations:** 1 The First Affiliated Hospital of Xinxiang Medical University, Weihui, Henan 453100;; 2 Department of Laboratory Medicine, Xinxiang Medical University, Xinxiang, Henan 453003, P.R. China

**Keywords:** tuberculosis, interferon-γ release assay, *Mycobacterium tuberculosis*

## Abstract

The aim of this study was to evaluate the sensitivity and specificity of a whole blood interferon-γ release assay, the QuantiFERON^®^-TB Gold In-Tube (QFT-GIT) test, in the diagnosis of *Mycobacterium tuberculosis* (MTB) infection, and to assess its monitoring role during antitubercular treatment. In total, 20 patients received the QFT test, along with other commonly-used tests, prior to, and following, 2- and 6-month courses of antitubercular treatment; the results were compared and statistically analyzed. The rate of positive results for tuberculosis (TB) was 95% for the QFT test, which was significantly higher compared with those for the purified protein derivative (PPD; 55%) and the antitubercular antibody tests (15%), as well as the acid-fast bacilli smear (20%) and cultures for TB (20%; P<0.05 for all). The sensitivity and specificity of the QFT test were 96 and 93.8%, respectively. The positive result rate obtained with the QFT test was significantly higher in the TB group compared with that in the non-TB group (6.3%; P<0.05). Moreover, the positive result rate obtained with the QFT test was significantly lower in the 6-month-treated group compared with that in the 2-month group (P<0.05). In conclusion, the QFT test is a sensitive and specific method for rapidly diagnosing MTB infection, and has an improved practical clinical value in evaluating antitubercular therapies compared with that of the PPD test.

## Introduction

Tuberculosis (TB) is a common infectious disease caused by the bacterium *Mycobacterium tuberculosis* (MTB), which continues to pose a serious threat to human life worldwide. The rapid and accurate diagnosis of MTB-infected individuals, or patients with TB, is the focus of TB control (1). The tuberculin skin test (TST) and acid-fast bacilli (AFB) sputum smear test have been widely used in the clinical diagnosis of TB. However, the specificity of the TST is poor, owing to its cross-reactivity with the Baccillus Calmette-Guérin (BCG) vaccination: Furthermore, there is a low positive result rate in the AFB sputum smear test. A positive bacteriological examination is the gold standard for diagnosis of TB; however, it is a slow procedure, which may prevent a timely diagnosis in the clinic. The development of the MTB-specific interferon-γ (IFN-γ) release assay (IGRA) has been considered to be a breakthrough for the diagnosis of TB infections (2,3). At present, there are two commercially available IGRA kits: the QuantiFERON^®^-TB Gold In-Tube (QFT-GIT) test (Cellestis Ltd., Carnegie, Australia), and the T-SPOT^®^.*TB* test (Oxford Immunotec, Abingdon, UK) (4). A previous study has indicated that the specificity, sensitivity and the positive result rate are all higher in the T-SPOT.*TB* test than in the traditional purified protein derivative (PPD) test (5). In the present study, we compared different methods utilized in the diagnosis of TB, including the QFT test, the AFB sputum smear, the TB antibody detection test and the PPD test, in the diagnosis of patients with active TB, as well as patients without TB. In addition, we performed the QFT test in the treatment process of the patients with active TB, and evaluated its role in the clinical diagnosis and treatment of TB.

## Subjects and methods

### Subjects

All subjects recruited to the study were diagnosed by our hospital (The First Affiliated Hospital of Xinxiang Medical University, Weihui, China), in accordance with the criteria developed by the Tuberculosis Branch of the Chinese Medical Association (Beijing, China). The subjects included 20 cases with positive MTB culture results (11 males and 9 females; age range, 18–72 years; mean age, 37.8±30.23 years; TB group), and 16 with negative MTB culture results (5 males and 11 females; age range, 21–49 years; mean age, 33.5±19.7 years; non-TB group). All participants were negative for HIV antibodies. The study was conducted in accordance with the Declaration of Helsinki, and with approval from the ethics committee of Xinxiang Medical University. Written informed consent was obtained from all participants.

### Whole blood IFN-γ assays

Peripheral venous blood samples were collected from each patient, and assayed with a QFT-GIT test kit (Cellestis Ltd.), in accordance with the manufacturer’s instructions. In brief, 1 ml heparinized blood was added to three tubes, containing the positive and negative controls, and the TB antigen, respectively, within 6 h. These were then incubated for 24 h at 37°C. The serum was harvested by centrifugation and used for IFN-γ enzyme-linked immunosorbent assay (ELISA). The results were subsequently analyzed with A-QFT software (Cellestis Ltd.), using the following interpretive criteria based on TB antigen response (TAR): positive, TAR(TB antigen tube-negative control tube) ≥0.35 IU/ml; negative, TAR(positive control tube) >0.5 IU/ml and TAR(TB antigen tube-negative control tube) <0.35 IU/ml; and uncertain, TAR(positive control tube) <0.35 IU/ml and TAR(TB antigen tube-negative control tube) <0.35 IU/ml, or TAR(negative control tube) ≥0.8 IU/ml

### TB antibody test

Fasting venous blood (3–5 ml) was collected from each patient, and the serum was separated by centrifugation. TB antibodies were detected by a colloidal gold method kit (Shanghai Aopu Biotechnology Co., Ltd., Shanghai, China) in accordance with the manufacturer’s instructions.

### PPD test

Subjects were injected intradermally with 0.1 ml tuberculin PPD, containing five units of tuberculin, into the anterior region of the forearms. The skin response was assessed 72 h later, and ≥10 mm of firm swelling at the site was considered to be a positive result.

### Anti-TB treatment

Patients with TB were administered with a combination treatment, including anti-TB drugs, such as isoniazid, as well as adjuvant nutritional support.

### Statistical analysis

Data were analyzed using SPSS software (SPSS, Inc., Chicago, IL, USA). The positive result rates were compared using a χ^2^ test, whilst the levels of IFN-γ release were analyzed using the non-parametric Mann-Whitney test. P<0.05 was considered to indicate a statistically significant difference.

## Results

### Comparison of the QFT test with conventional tests in the diagnosis of MTB infection

For the patients with TB, the positive result rate in the QFT test was significantly higher than those in the AFB smear, TB antibody, bacterial culture and PPD tests (all P<0.05; Table I).

### Evaluation of the QFT, PPD and TB antibody tests in patients with and without TB

As demonstrated in Table II, the QFT test was positive in 19 out of 20 TB cases, with a sensitivity of 96%, and negative in 15 out of 16 non-TB cases, with a specificity of 93.8%. The PPD test exhibited a sensitivity and specificity of 54% (11/20) and 75% (12/16), respectively (Table III). In addition, the sensitivity and specificity of the TB antibody test were 15% (3/20) and 87.5% (14/16), respectively (Table IV). The comparative analysis revealed that the sensitivity and specificity of the QFT test were significantly improved compared with those of the PPD and TB antibody tests (both P<0.05).

### Effects of the course of anti-TB treatment on the tests for TB

In total, 20 patients with TB received the QFT test, following 2- and 6-month courses of anti-TB treatment. The results demonstrated that the positive result rate for the QFT test was significantly lower in the 6-month treatment group than that in the 2-month treatment group (P<0.05). However, no significant difference was observed between the two groups in the PPD test (Table V).

## Discussion

The MTB-specific IGRA represents a significant advance in the diagnosis of TB infections. The mechanism of this test is based on the principle that sensitized T cells, which are produced as a result of exposure to MTB antigens, secrete IFN-γ when they become re-exposed to similar antigens. Therefore, when stimulated by MTB-specific antigens, the infected blood releases IFN-γ. Thus, patients with TB may be identified by IFN-γ detection in the blood as a whole, or in the mononuclear cells isolated from peripheral blood samples. The QFT test measures the levels of IFN-γ released from peripheral blood lymphocytes by ELISA. The antigens used in the assay are the 6 kDa early secreted antigenic target (ESAT-6) of MTB, and the 10 kDa culture filtrate protein (CFP-10), which are specific to MTB (6–8), and exhibit little antigenic cross-reactivity with the BCG vaccination or environmental nontuberculous mycobacteria. Thus, this method is highly sensitive and specific, and has been approved for clinical application in a number of locations, including the United States, the European Union, Canada and Japan.

In the present study, the positive result rate for MTB was 95% (19/20) in the QFT test, which was significantly higher than those in the conventional tests, including the PPD, TB antibody, mycobacterial culture and AFB smear tests (P<0.01 for all). Moreover, the sensitivity and specificity of the QFT test were improved in comparison with the clinically-used TB antibody and PPD tests (P<0.05). These results suggest that the QFT test has an important clinical significance in the diagnosis of patients with TB. A number of the patients with TB did not exhibit any clinical features, whilst several patients presented with extrapulmonary tuberculosis. Therefore, the clinical manifestations of TB were complicated, dormant and not typical. The conventional tests, including the PPD, TB antibody tests and AFB smear tests, did not demonstrate sufficient sensitivity or specificity, whilst the mycobacterial culture test was slow. These tests have been demonstrated to lead to missed and inaccurate diagnoses, and, as a result, a delay in treatment (9,10). Thus, these diagnostic tests are limited in clinical practice. By contrast, the QFT test demonstrated an improved sensitivity and specificity, and only required 2 days for the diagnosis. Therefore, it was particularly beneficial in the diagnosis of the patients with subclinical or unclear infections, enabling early diagnosis and treatment.

The first two months of TB treatment are known as the intensive phase. This study compared the positive result rates of the QFT and the conventional tests following 2- and 6-month periods of treatment. It was demonstrated that the positive result rate of the QFT test was significantly lower in the 6-month treatment group than that in the 2-month group, indicating that this test was more effective than the traditional skin test in the process of initial diagnosis. However, there was little difference between the positive result rates of the PPD and TB antibody tests during the treatment. Furthermore, the positive result rates approached zero in the AFB smear and the MTB culture tests, predominantly due to the reduction in the internal levels of bacteria as the therapy progressed. Additionally, the proliferation of peripheral mononuclear cells and the secretion of IFN-γ were promoted by ESAT-6 and CFP-l0, two MTB-specific protein antigens, in the patients with MTB infection. However, IFN-γ is secreted solely by effector memory T cells (11,12). Notably, these cells are only present in the body whilst it is infected with MTB; their levels are reduced by treatment and eliminated when the infection is completely cured. Therefore, the QFT test is only positively correlated with the levels of active MTB in the body, and is unaffected by past infections. As a result, the QFT test is an ideal indicator for assessing the efficacy of anti-TB treatments.

At present, the provisional diagnosis of TB due to MTB infection is based on the PPD test; however, the PPD test demonstrates an extensive cross-reactivity with the BCG vaccination and a number of the environmental nontuberculous mycobacteria (13–15). We conclude that, in comparison with the conventional diagnostic tests, the whole-blood QFT test demonstrated improved rapidity and sensitivity. This suggests its clinical potential, not only in the diagnosis of TB, but also in the evaluation of the efficacy of anti-TB treatments.

## Figures and Tables

**Table I.** Positive results for different tests in the diagnosis of MTB infection.

Group	n	PPD n (%)	TB antibody n (%)	AFB smear n (%)	MTB culture n (%)	QFT n (%)
Patients with TB	20	11 (55)	3 (15)	4 (20)	4 (20)	19 (95)
Non-TB patients	16	4 (25)	2 (12.5)	0 (0)	0 (0)	1 (6.3)

MTB, *Mycobacterium tuberculosis*; PPD, purified protein derivative; TB, tuberculosis; AFB, acid-fast bacilli; QFT, QuantiFERON^®^-TB.

**Table II.** Evaluation of the QuantiFERON^®^-TB Gold In-Tube (QFT-GIT) test in the detection of MTB in patients with TB.

Group	n	Positive (n)	Negative (n)	Positive rate (%)	Specificity (%)	Sensitivity (%)	Negative predictive value (%)
Patients with TB	20	19	1	95.0	-	96	93
Non-TB patients	16	1	15	6.3	93.8	-	-

MTB, *Mycobacterium tuberculosis*; TB, tuberculosis.

**Table III.** Evaluation of the PPD test in the detection of MTB in patients with TB.

Group	n	Positive (n)	Negative (n)	Positive rate (%)	Specificity (%)	Sensitivity (%)	Negative predictive value (%)
Patients with TB	20	11	9	55	-	54	74.8
Non-TB patients	16	4	12	25	75	-	-

PPD, purified protein derivative; MTB, *Mycobacterium tuberculosis*; TB, tuberculosis.

**Table IV.** Evaluation of the TB antibody test in the detection of MTB in patients with TB.

Group	n	Positive (n)	Negative (n)	Positive rate (%)	Specificity (%)	Sensitivity (%)	Negative predictive value (%)
Patients with TB	20	3	17	15.0	-	15	87
Non-TB patients	16	2	14	12.5	87.5	-	-

TB, tuberculosis; MTB, *Mycobacterium tuberculosis*.

**Table V.** Positive result rates for different tests for TB during anti-TB treatment.

Treatment group	n	PPD n (%)	TB antibody n (%)	AFB smear n (%)	MTB culture n (%)	QFT n (%)
2 months	20	11 (55)	3 (15)	4 (20)	4 (20)	19 (95)
6 months	20	10 (50)	4 (20)	2 (10)	0 (0)	6 (30)

TB, tuberculosis; PPD, purified protein derivative; AFB, acid-fast bacilli; MTB, *Mycobacterium tuberculosis;* QFT, QuantiFERON^®^-TB.
